# Taxonomic and faunistic studies on the genus *Harutaeographa* (Lepidoptera, Noctuidae, Orthosiini) with description of a new species

**DOI:** 10.3897/zookeys.242.3889

**Published:** 2012-11-15

**Authors:** Balázs Benedek, Aidas Saldaitis, Jolanta Rimsaite

**Affiliations:** 1 H-2045 Törökbálint, Árpád u. 53, Hungary; 2 Nature Research Centre, Akademijos str. 2, LT–08412 Vilnius-21, Lithuania; 3 Nature Research Centre, Akademijos str. 2, LT–08412 Vilnius-21, Lithuania

**Keywords:** Orthosiini, new species, *Harutaeographa*, distribution

## Abstract

Description of anew *Harutaeographa* Yoshimoto, 1993 species, *Harutaeographa shui* **sp. n.** from China’s Sichuan province, is given. *Harutaeographa yangzisherpani transformis* Hreblay & Ronkay, 1999 is combined as a synonym of *Harutaeographa yangzisherpani yangzisherpani* Hreblay & Ronkay, 1999. Additional distributional data for *Harutaeographa pallida* Yoshimoto, 1993, and *Harutaeographa cinerea* Hreblay & Ronkay, 1998 are provided. A checklist of the genus *Harutaeographa* and a key to the *Harutaeographa fasciculata* (Hampson, 1894) species-group, based on external characters and genitalia, are presented.

## Introduction

This paper contributes additional taxonomic, genitalic and faunistic information on the taxonomy of the genus *Harutaeographa* Yoshimoto, 1993 to what was previously provided in Chen (1999), Hacker and Ronkay (1996), Hampson (1894, 1906), Hreblay and Ronkay (1998, 1999), Hreblay et al. (1998), Kononenko et al. (1998), Ronkay and Ronkay (2000), Ronkay et al. (2001, 2010), and Yoshimoto (1993, 1994). The genus *Harutaeographa* is typical of the tribe *Orthosiini* within the Himalayan Noctuidae and contains 37 species and 3 subspecies distributed mostly in the Southeast Asian and Himalayan regions. Except for a few Western-Himalayan and Central-Asian species inhabiting semi-dry areas, most members of this genus are associated with the Himalayan monsoonic forest belt. Flight periods generally extend through March and April, but some Southern-Himalayan species are on the wing during the colder November to February period.

## Materials and methods

The specimens of *Harutaeographa* preserved in the collections of Alessandro Floriani (Milan, Italy), Balázs Benedek (Törökbálint, Hungary), Gottfried Behounek (Grafing, Germany)/Zoologische Staatssammlung, Munich (Germany), Danny Nilsson (Kalvehave, Denmark) and Nature Research Centre (Vilnius, Lithuania) were examined. The specimens examined were collected in China and Nepal using ultraviolet light traps and occasionally sugar ropes. Seventeen genital slides were prepared and 27 photographs were made. Examination of morphology: after maceration, male and female genitalia were dissected and mounted in euparal on glass sides. Dissection of genitalia follows Lafontaine (2004). Photographs of genitalia were made using a Wild M3Z microscope and Canon EOS 350D camera.

Abbreviations of the material depositories:

AFMAlessandro Floriani (Milan, Italy);


BBTBalázs Benedek (Törökbálint, Hungary);


BMNHNatural History Museum, London (United Kingdom);


DNKDanny Nilsson (Kalvehave, Denmark);


HNHMHungarian Natural History Museum, Budapest (Hungary);


MNHUMuseum für Naturkunde Leibniz Institute for Research on Evolution and Biodiversity, Berlin (Germany);


NHMNaturhistorisches Museum Wien (Austria);


NRCVNature Research Centre (Vilnius, Lithuania);


NSMTNational Science Museum Tokyo (Japan);


ZFMKZoological Forschungsinstitut und Museum Alexander Koenig, Bonn (Germany);


ZMHZoological Museum of Helsinki (Finland);


ZSMZoologische Staatssammlung, Munich (Germany).


## Systematic accounts

**Key to *Harutaeographa* species related to *Harutaeographa fasciculata* based on external characters**

**Table:** 

1	Forewings dark brown with black pattern or patches of dark scales	2
	Forewings light brown, orbicular stigma whitish (Figs 10, 11)	*Harutaeographa fasciculata* (Himalaya: Sikkim, N.India; Nepal)
2	Wingspan of forewings 37–42 mm, forewings narrow, cilia golden yellow (Figs 6, 7)	*Harutaeographa shui* (China: Sichuan)
	Wingspan of forewings 42–48 mm, forewings wide, cilia dark brown (Figs 8, 9)	*Harutaeographa odavissa* (China: Shaanxi, Hubei, Sichuan)

**Key to *Harutaeographa* species related to *Harutaeographa fasciculata* based on genital characters**

**Table:** 

1	Valva at apex widening, clasper curved, ductus bursae straight	2
–	Valva not widening at apex, clasper almost straight, ductus bursae curved at base (Figs 17, 23)	*Harutaeographa fasciculata*
2	Uncus short, apical part of cucullus a boot-shaped plate with short blunt apex, ampula long, curved at apex, almost perpendicular to costa, both anterior and posterior parts of corpus bursae almost equal in size (Figs 15, 21)	*Harutaeographa shui* sp. n.
–	Uncus 1/3 longer than in *Harutaeographa shui*, apical part of cucullus boot-shaped with short acute apex, ampula slightly curved at apex, posterior part of corpus bursae longer and narrower than anterior part (Figs 16, 22)	*Harutaeographa odavissa*

### Harutaeographa shui

Benedek & Saldaitis sp. n.

urn:lsid:zoobank.org:act:C3F72129-2643-4234-84F6-ABEA933B13A8

http://species-id.net/wiki/Harutaeographa_shui

Figs 6, 7 15 21

#### Type material.

**Holotype**:male (Fig. 6),China, Sichuan, 29°43.105'N, 02°36.195'E, near Siping, 1600 m, 27.iii.2011, Floriani leg., in the collection of ZSM; (slide No. JB1792m).

**Paratypes**: 3 males, with the same data as the holotype, 1 male, from the same locality, but 02.iv.2011, 1 female (Fig. 7), China, W. Sichuan, road Menghugang/Kangding, 29°49.955'N, 102°02.827'E, 1500 m, 19.iv.2010, leg. Chen Gun, in the AFM, BBT, and ZSM collections. Slide No. JB1793f.

#### Etymology.

The specific name refers to the Shu Kingdom, which is now Chengdu, the capital of China’s Sichuan province.

#### Diagnosis.

*Harutaeographa shui* in general appearance is similar to *Harutaeographa fasciculata* (Hampson, 1894) (Figs 10, 11), but is smaller and has more oblong forewings. Despite this superficial resemblance to *Harutaeographa fasciculata*, based on genital morphology the new species is more closely related to *Harutaeographa odavissa* Ronkay, Ronkay, Gyulai & Hacker, 2010 (Figs 8, 9). These species are easily distinguishable externally by forewing shape, coloration and pattern. The male genitalia differ from those of *Harutaeographa odavissa* (Fig. 16)by its shorter uncus, smaller tegumen, remarkably broader and apically more elongate cucullus, and the configuration of the clasper-ampulla complex. The structure of aedeagus and vesica are similar to those of *Harutaeographa odavissa* (Fig. 16), but *Harutaeographa shui* (Fig. 15) has a slightly more curved aedeagus, differently configured vesica, and longer subterminal cornuti field. The female genitalia of the new species differ from those of *Harutaeographa odavissa* (Fig. 22) in having a shorter ovipositor, shorter apophyses, and shorter and weaker ductus bursae.

#### Description.

Wingspan 37–42 mm, length of forewing 17–20 mm. Head, front and thorax chocolate brown with some copper shine; male antenna bipectinate, female antenna narrow ciliate; forewings richly decorated with dark coppery-brown patterns distinctly marked with black scales, outer margin and cilia lighter golden yellow; hindwings with intensive dark suffusion, especially wide on outer margin, discal spot, and well-marked postmedial fascia; cilia with copper shine. **Male genitaila** (Fig. 15): uncus short, evenly broad; tegumen small and low; vinculum strong, narrow, V-shaped; valva finely arcuate; cucullus broad with apex elongate; sacculus weak, less sclerotized; clasper and ampulla robust outside and turned at middle; aedeagus rather long, gently arcuate; vesica with two subbasal coils and a small rasp-plate at base near carina, two small bunches of stronger cornuti in subbasal coils, and a long brush-like cornuti field on arcuate subterminal area. **Female genitalia** (Fig. 21): ostium nearly evenly truncated; ductus bursae narrow, somewhat wider and less sclerotized posteriorly; appendix bursae relatively small and rounded, weakly sclerotized; corpus bursae elongated, mesially constricted, with anterior and posterior parts subequal.

#### Bionomics and distribution.

The new species is known only from Siping and Kangding areas of Sichuan Province (China), on the eastern edge of Tibetan plateau, where a few specimens were collected at the end of March – beginning of April at altitudes ranging from 1500 to 1600 m. It was attracted to light during cold (2–4 ˚C) nights in small river valleys. The habitat is mountain virgin mixed forest dominated by various broad-leaved trees, rhododendrons and bamboos (Fig. 27).

**Figures 1–5.**
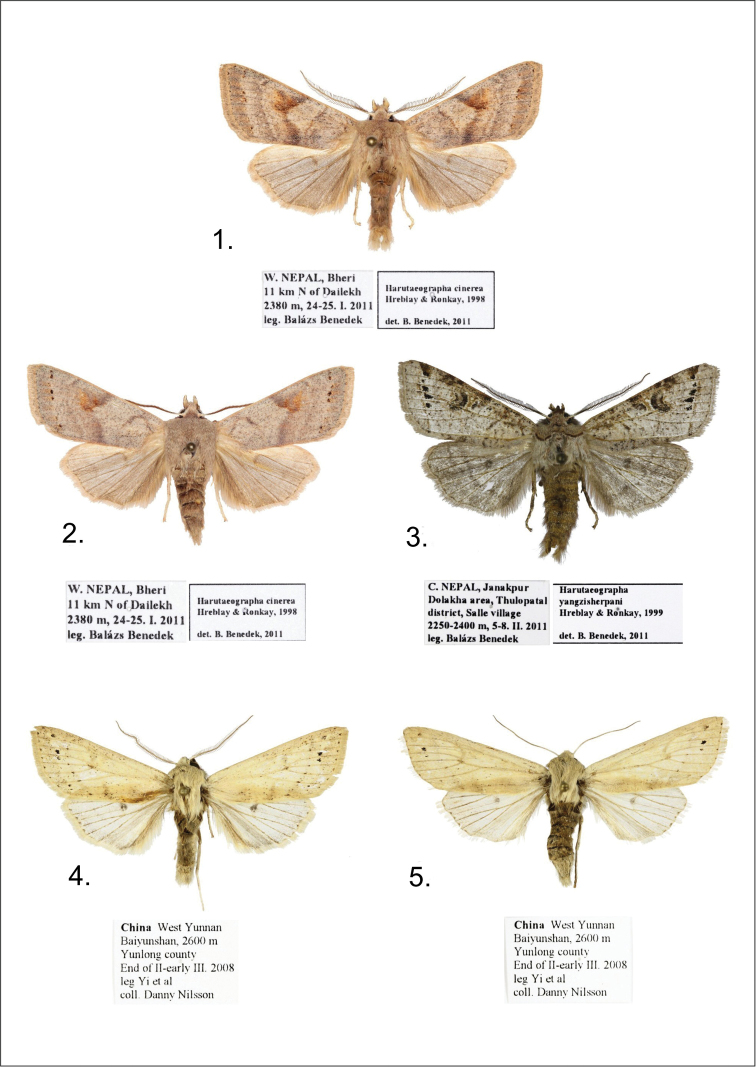
*Harutaeographa* ssp. adults. **1** *Harutaeographa cinerea*, male, West-Nepal, Bheri (BBT) **2** *Harutaeographa cinerea*, female, West-Nepal, Bheri (BBT) **3** *Harutaeographa yangzisherpani yangzisherpani*, male, Nepal, Janakpur (BBT) **4** *Harutaeographa monimalis*, male, China, W. Yunnan (DNK) 5 *Harutaeographa monimalis*, female, China, W. Yunnan (DNK)

**Figures 6–13.**
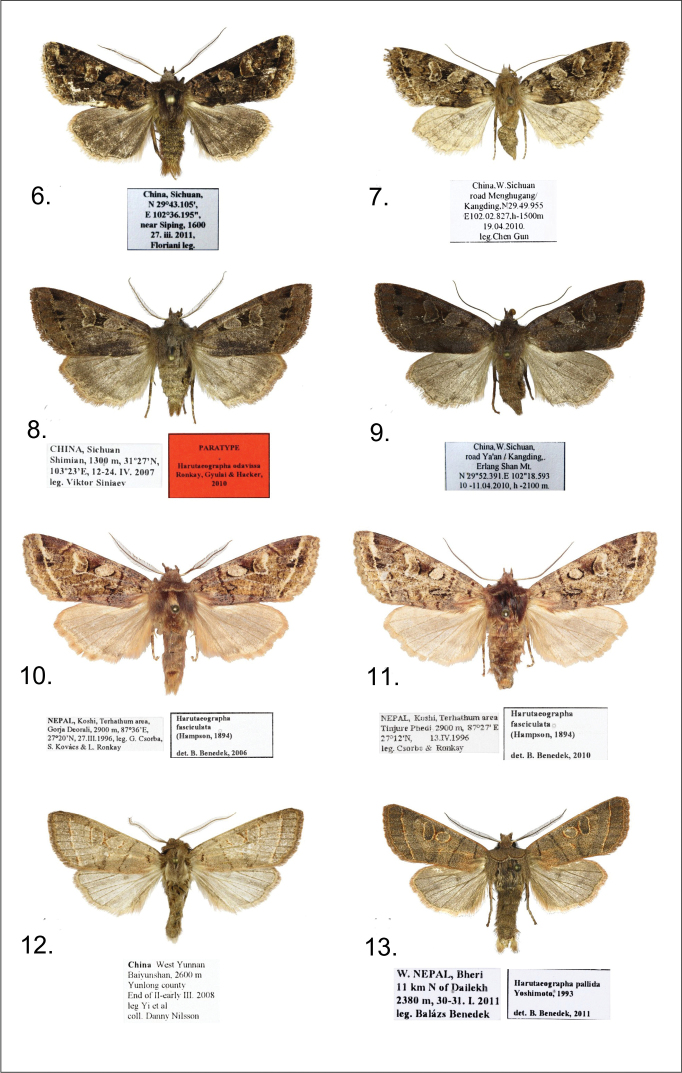
*Harutaeographa* ssp. adults. **6** *Harutaeographa shui*, male, holotype, China, Sichuan (GBG/ZSM) **7** *Harutaeographa shui*, female, paratype, China, Sichuan (AFM) **8** *Harutaeographa odavissa*, male, paratype, China, Sichuan (BBT) **9** *Harutaeographa odavissa*, female, China, Sichuan (BBT) **10** *Harutaeographa fasciculata*, male, Nepal, Koshi (BBT) **11** *Harutaeographa fasciculata*, female, Nepal, Koshi (BBT) **12** *Harutaeographa pallida*, male, China, W. Yunnan (DNK) **13** *Harutaeographa pallida*, male, West-Nepal, Bheri (BBT)

### Harutaeographa monimalis

(Draudt, 1950)

http://species-id.net/wiki/Harutaeographa_monimalis

Figs 4, 5 18 24

#### Material examined.

1 male, 1 female, China, W. Yunnan, Baiyunshan, 2600 m, Yunlong county, end of ii - early iii.2008, leg. Yi et al, in the collection of DNK; slide Nos JB1851m (Fig. 18); JB1852f.

#### Diagnosis.

**Male genitalia.** Uncus small, elongated; tegumen short; juxta long, tongue-shaped, more or less quadrangular; vinculum short and heavily sclerotised, U-shaped; sacculus broad, with more or less parallel margins. Clasper relatively large, thumb-like, with elongated and heavily sclerotised base; clasper fused with relatively small and evenly curved ampulla. Valvae more or less symmetrical, broad, armed with strong, finger-shaped ventral process and large, broad digitus; cucullus broad and strong, more or less rhomboidal in shape. Aedeagus relatively long, straight and broad; vesica evenly helicoid in shape, everted ventrally, covered with a row of fine spiculi from basal part of vesica along to terminal segment where it merges into a stouter cluster of longer spines forming a brush-like structure. **Female genitalia** (Fig. 24): Ostium bursae wide, rounded; ductus relatively short; appendix bursae helicoid with three coils; corpus bursae constricted mesially with both anterior and posterior sections broadly elliptical.

#### Note.

This is the first new report of this species known previously only by its holotype specimen collected by Dr. H. Höne in 1935 (coll. ZFMK, Bonn).

### Harutaeographa yangzisherpani transformis

Hreblay & Ronkay, 1999 syn. nov.

http://species-id.net/wiki/Harutaeographa_yangzisherpani_transformis

Figs 3 19

#### Material examined.

2 males, Nepal, Janakpur, Dolakha area, Thulopatal district, Salle village, 2250–2400 m, 27°35.998'N, 86°09.775'E 5–8.ii.2011, leg. Balázs Benedek, in the collection of BBT; slide No. JB1807m (Fig. 19).

#### Note.

The genitalia of *Harutaeographa yangzisherpani yangzisherpani* Hreblay & Ronkay, 1999 from Nepal exactly match those of *Harutaeographa yangzisherpani transformis*Hreblay & Ronkay from N. Vietnam pictured in Esperiana, 1999. The very slight genital differences between*Harutaeographa yangzisherpani yangzisherpani* Hreblay & Ronkay, 1999 and *Harutaeographa yangzisherpani transformis*Hreblay & Ronkay, 1999, suggest that they representing the same taxon and it is not reasonable to separate them as subspecies. Thus, *Harutaeographa yangzisherpani transformis*Hreblay & Ronkay, 1999 is combined here as a synonym of *Harutaeographa yangzisherpani yangzisherpani* Hreblay & Ronkay, 1999. It is first report of this species from Nepal.

**Figures 14–18.**
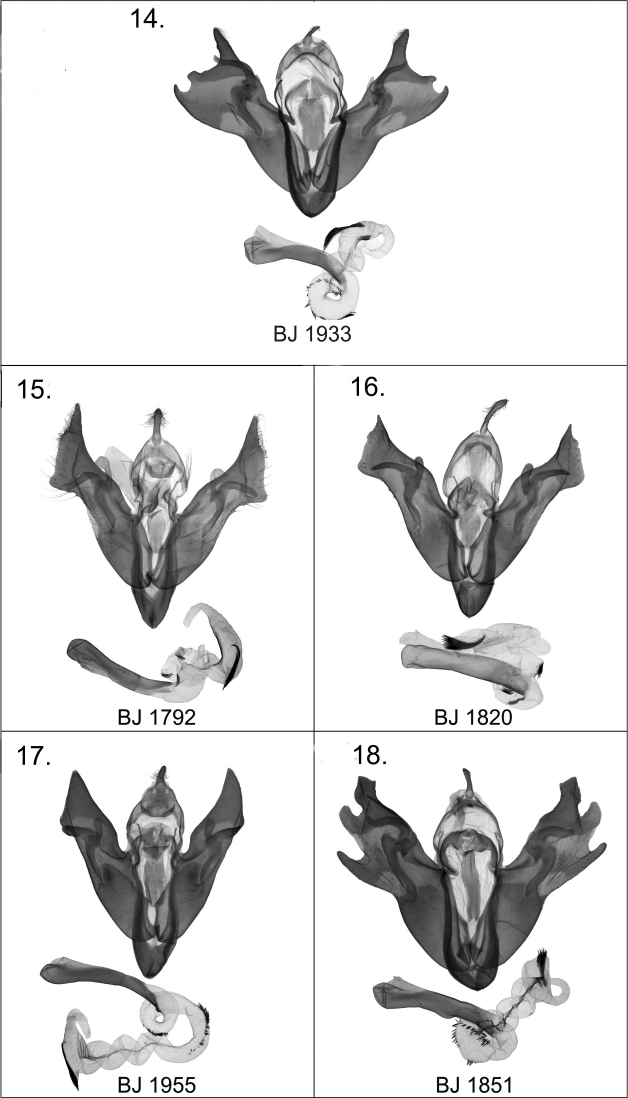
*Harutaeographa* ssp. male genitalia. **14** *Harutaeographa cinerea*, male, prep. BJ1933m **15** *Harutaeographa shui*, male, holotype, prep. BJ1792m **16** *Harutaeographa odavissa*, male, paratype, prep. BJ1820m **17** *Harutaeographa fasciculata*, male, prep. BJ1955m **18** *Harutaeographa monimalis*, male, prep. BJ1851m

**Figures 19–26.**
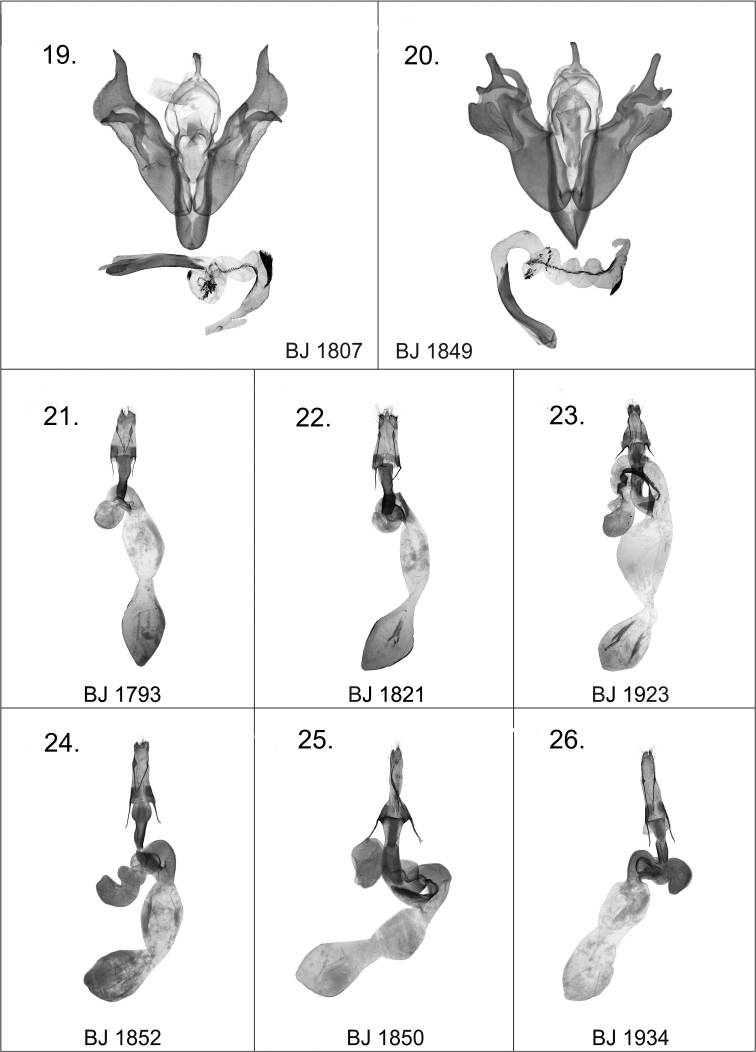
*Harutaeographa* ssp. male and female genitalia. **19** *Harutaeographa yangzisherpani yangzisherpani*, male, prep. BJ1807m **20** *Harutaeographa pallida*, male, prep. BJ1849m **21** *Harutaeographa shui*, female, paratype, prep. BJ1793f **22** *Harutaeographa odavissa*, female, prep. BJ1821f **23** *Harutaeographa fasciculata*, female, prep. BJ1923f **24** *Harutaeographa monimalis*, female, prep. BJ1852f **25** *Harutaeographa pallida*, female, prep. BJ1850f **26** *Harutaeographa cinerea*, female, prep. BJ1934f

### Harutaeographa pallida

Yoshimoto, 1993

http://species-id.net/wiki/Harutaeographa_pallida

Figs 12, 13 20, 25

#### Material examined.

1 female, West-Nepal, Bheri, Dailekh area, 11 km N of Dailekh, 2380 m, 24–25.i.2011, 1 male, West-Nepal, Bheri, Dailekh area, 11 km N of Dailekh, 2380 m, 30–31.i.2011, 2 males, Nepal, Janakpur, Dolakha area, Thulopatal district, Salle village, 2250–2400 m, 5–8.i.2011, leg. Balázs Benedek, 3 males, 1 female, China, W. Yunnan, Baiyunshan, 2600 m, Yunlong county, end of ii - early iii.2008, leg. Yi et al, in the collections of BBT and DNK; slide Nos JB1849m, JB1850f, (Figs 20, 25).

#### Notes.

These specimens, which are paler in colour than those from Nepal, are the first to be reported from China.

### Harutaeographa cinerea

Hreblay & Ronkay, 1998

http://species-id.net/wiki/Harutaeographa_cinerea

Figs 1, 2 14 26

#### Material examined.

long series of both sexes from the following localities: West-Nepal, Bheri, Dailekh area, 11 km N of Dailekh, 2380 m, 24–25.i.2011, West-Nepal, Bheri, Dailekh area, 13 km N of Dailekh, 2425 m, 26–27.i.2011, West-Nepal, Bheri, Dailekh area, 12 km N of Dailekh, 2600 m, 29.i.2011, West-Nepal, Bheri, Dailekh area, 11 km N of Dailekh, 2380 m, 30–31.i.2011, Nepal, Janakpur, Dolakha area, Thulopatal district, Salle village, 2250–2400 m, 5-8.ii.2011, leg. Balázs Benedek, in the collection of BBT.

#### Note.

Little information has been available for this species due to insufficient collecting during its very early flight period (Hreblay and Ronkay 1998; Hreblay and Ronkay 1999). At the end of January–beginning of February in 2011 *Harutaeographa cinerea* was one of the most frequently encountered noctuid species at light and sugar ropes at elevations between 2300–2600 m and is presumably widespread in Nepal.

**Figure 27.**
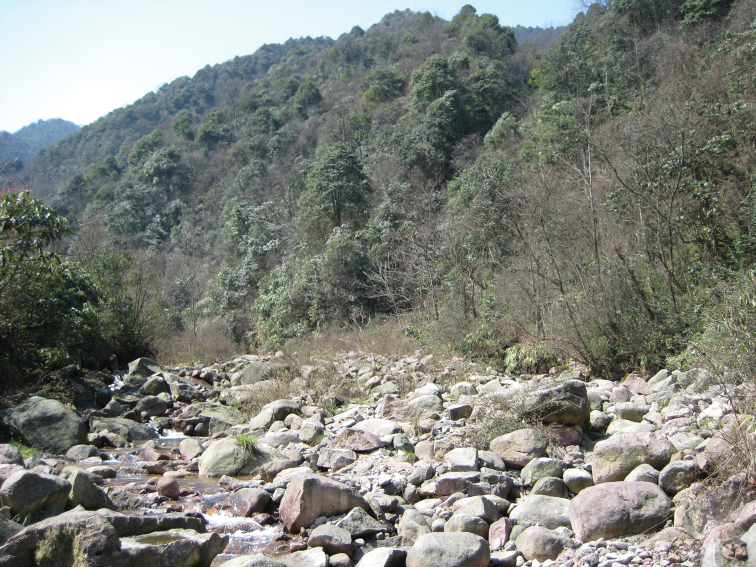
*Harutaeographa shui*,type locality, China, Sichuan near Siping.

### Checklist of the genus *Harutaeographa*

***Harutaeographa adusta* Hreblay & Ronkay, 1999**

Holotype: coll. M. Hreblay, HNHM. Type locality: Thailand, Changwat Chiang Mai, Mt. Doi Inthanon. Distribution: Indochina, Thailand.

***Harutaeographa akos* Hreblay, 1996 [1997]**

Holotype: ZMH. Type locality: Tadjikistan, Gissar-Gebirge, Romit. Distribution: Tadjikistan (Gissar Mts.).

***Harutaeographa babai* Sugi & Sakurai, 1994**

Holotype: NSMT. Type locality: Nepal, Dhaulagiri, Jomsom. Distribution: Nepal; Himalaya.

***Harutaeographa bidui bidui* Hreblay & Plante, 1996 [1997]**

Holotype: coll. M. Hreblay, HNHM. Type locality: N.Pakistan, 5 km S Rattu. Distribution: Himalaya: N. Pakistan (Karakorum, Prov. Gilgit & Baltistan, Rattu, Prov. Jammu & Kashmir, Deosai Mts.).

***Harutaeographa bidui kaghanensis* Hreblay & Ronkay, 1999**

Holotype: coll. Hreblay, HNHM. Type locality: Pakistan, Prov. NW-Frontier, Kaghan valley, Khanian. Distribution: Pakistan (Prov. NW-Frontier, Kaghan valley, Khanian).

***Harutaeographa bicolorata* Hreblay & Ronkay, 1998**

Holotype: coll. Hreblay, HNHM. Type locality: Nepal, Ganesh Himal, 1 km E Gadrang. Distribution: Himalaya: Nepal (Ganesh Himal).

***Harutaeographa brahma* Hreblay & Ronkay, 1998**

Holotype: coll. Hreblay, HNHM. Type locality: Nepal, Ganesh Himal, 2 km W Thangjet. Distribution: Himalaya: Nepal (Ganesh Himal).

***Harutaeographa brumosa* Yoshimoto, 1994**

Holotype: NSMT. Type locality: Nepal, Janakpur, Jiri. Distribution: Himalaya: Nepal (Janakpur, Jiri, Terhathum distr.).

***Harutaeographa caerulea caerulea* Yoshimoto, 1993**

Holotype: NSMT. Type locality: Nepal, Mt. Phulchouki. Distribution: Himalaya: Nepal (Katmandu Valley, Godavari).

***Harutaeographa caerulea rubrigrapha* Hreblay & Ronkay, 1999**

Holotype: coll. Hreblay, HNHM. Type locality: Thailand, Changwat Chang Mai, Mt. Doi Phahompok, 18 km NW Fang. Distribution: Indochina: Thailand (Chiang Mai).

***Harutaeographa castanea* Yoshimoto, 1993**

Holotype: NSMT. Type locality: Nepal, Godavari. Distribution: Himalaya: Nepal (Kathmandu Valley, Godavari).

***Harutaeographa castaneipennis* (Hampson, 1894)**

Holotype: BMNH. Type locality: India, Kashmir, Narkundah. Distribution: Himalaya: N.India (Prov. Jammu & Kashmir).

***Harutaeographa cinerea* Hreblay & Ronkay, 1998**

Holotype: coll. G. Ronkay. Type locality: Nepal, Ganesh Himal, near Slya. Distribution: Nepal (Ganesh Himal, Slya).

***Harutaeographa craspedophora* (Boursin, 1969)**

Holotype: coll. Vartian, NHM. Type locality: Afghanistan, Paghman-Gebirge, 20 km NW Kabul. Distribution: Afghanistan (Paghman Mts.).

***Harutaeographa diffusa* Yoshimoto, 1994**

Holotype: NSMT. Type locality: Nepal, Janakpur, Jiri. Distribution: Himalaya: Pakistan; Nepal.

***Harutaeographa elphinia* Hreblay & Ronkay, 1999**

Holotype: HNHM. Type locality: Vietnam, Prov. Lao Cai, Mt. Fan-si-Pan, 7 km SW Sa Pa. Distribution: Indochina, Vietnam (Prov. Lao Cai, Tonkin).

***Harutaeographa eriza* (Swinhoe, 1901)**

Holotype: BMNH. Type locality: W. India, Punjab, Himachal Pradesh, Kulu. Distribution: Himalaya; Pakistan; India (Prov. Punjab).

***Harutaeographa fasciculata* (Hampson, 1894)**

= *Harutaeographa fusciculata* nec Hampson, 1894

Holotype: BMNH. Type locality: Sikkim (India). Distribution: Himalaya: Sikkim, N. India; Nepal; North Vietnam (Fansipan Mts).

***Harutaeographa ferrosticta* (Hampson, 1894)**

Holotype: BMNH. Type locality: Kashmir, Narkundah. Distribution: Himalaya: Pakistan; N. India (Prov. Jammu & Kashmir).

***Harutaeographa ganeshi* Hreblay & Ronkay, 1998**

Holotype: coll. G. Ronkay. Type locality: Nepal, Ganesh Himal, 2 km W Gholjong. Distribution: Himalaya: Nepal (Ganesh Himal, Gholjong).

***Harutaeographa izabella* Hreblay & Ronkay, 1998**

Holotype: coll. Hreblay, HNHM. Type locality: Nepal, Annapurna Himal, 1 km E Ghorepani. Distribution: Nepal.

***Harutaeographa kofka* Hreblay, 1996 [1997]**

Holotype: BMNH. Type locality: N. India, Muktesar, Naini-Tal. Distribution: Himalaya: Pakistan; N. India; Nepal.

***Harutaeographa loeffleri* Ronkay, Ronkay, Gyulai & Hacker, 2010**

Holotype: coll. P. Gyulai, HNHM. Type locality: Burma, Chun state, Mindat camp. Distribution: Myanmar (Chun state, Mindat camp, Chun state, Natmataung Nationalpark, Mt. Victoria).

***Harutaeographa maria* Hreblay & Ronkay, 1999**

Holotype: coll. Hreblay, HNHM. Type locality: Pakistan, Prov. Jammu & Kashmir, Naltar valley, 5 km E Naltar. Distribution: Himalaya: Pakistan (Prov Jammu & Kashmir, Karakorum).

***Harutaeographa marpha* Hreblay & Ronkay, 1999**

Holotype: coll. Hreblay, HNHM. Type locality: Nepal, Dhaulagiri Himal, 6 km NW Marpha. Distribution: Nepal; Himalaya.

***Harutaeographa monimalis* (Draudt, 1950)**

Holotype: ZFMK. Type locality: China, Yunnan. Distribution: China (Prov. Yunnan).

***Harutaeographa odavissa* Ronkay, Ronkay, Gyulai & Hacker, 2010**

Holotype: HNHM. Type locality: China, Shaanxi, Taibaishan. Distribution: China (Prov. Shaanxi, Taibaishan, Tsinling Mts., Prov. Hubei, Daba Shan, Prov. Sichuan, Daxue Shan, Gongga Shan, Volong Reserve, Siguliang Shan, Qingcheng Shan).

***Harutaeographa orias orias* Hreblay, 1996 [1997]**

Holotype: BMNH. Type locality: Prov. W. Bengal, Darjeeling. Distribution: Himalaya: N.India (Prov. Sikkim, Prov. W. Bengal, Darjeeling).

***Harutaeographa orias yoshimotoi* Hacker & Hreblay, 1996 [1997]**

Holotype: coll. Hacker, ZSM. Type locality: N. India, Himachal Prad., Rohtang. Distribution: Pakistan (Prov. Kashmir), Himalaya: N. India (Himachal Pradesh, Rohtang Pass, Prov. Sikkim); Nepal; Indochina; Thailand (Prov. Chiang Mai, Doi Phahompok)

***Harutaeographa pallida* Yoshimoto, 1993**

Holotype: HNSMT, Tokyo (Japan). Type locality: Nepal, Godavari. Distribution: Himalaya: N. India (Prov. Sikkim); Nepal (Katmandu Valley, Godavari, Solu Khumbu Himal, Ganesh Himal); China (Prov. Yunnan).

***Harutaeographa pinkisherpani* Hreblay & Ronkay, 1998**

Holotype: coll. G. Ronkay. Type locality: Nepal, Ganesh Himal, 2 km SW Haku. Distribution: Himalaya: Nepal (Ganesh Himal, Haku).

***Harutaeographa rama* Hreblay & Plante, 1996 [1997]**

Holotype: coll. Hreblay, HNHM. Type locality: N.Pakistan, 10 km SW Astor, Rama. Distribution: Himalaya: Pakistan (Jammu & Kashmir).

***Harutaeographa rubida* (Hampson, 1894)**

= *Harutaeographa bipuncta* Yoshimoto, 1993

Holotype: BMNH. Type locality: Sikkim. Distribution: Himalaya: Nepal; N.India (Sikkim).

***Harutaeographa saba* Hreblay & Plante, 1996 [1997]**

Holotype: coll. M. Hreblay, HNHM. Type locality: N.Pakistan, 10 km SW Astor, Rama. Distribution: Pakistan; Afghanistan.

***Harutaeographa seibaldi* Ronkay, Ronkay, Gyulai & Hacker, 2010**

Holotype: coll. H. Seibald, Wien (Austria). Type locality: Burma, Chun state, Mindat camp. Distribution: Myanmar (Chun state, Mindat camp, Chun state, Natmataung Nationalpark, Mt. Victoria).

***Harutaeographa shui* Benedek & Saldaitis, 2012**

Holotype: ZSM. Type locality: China, Sichuan, 29°43.105'N, 102°36.195'E, near Siping. Distribution: China (Sichuan).

***Harutaeographa siva* Hreblay, 1996 [1997]**

Holotype: BMNH. Type locality: N. India, Simla. Distribution: Himalaya: N. India.

***Harutaeographa stangelmaieri* Ronkay, Ronkay, Gyulai & Hacker, 2010**

Holotype: coll. Becher/Stumpf (Germany). Type locality: China, Prov. Yunnan, Daxue Shan Mts. Distribution: China (Prov. Yunnan, Daxue Shan Mts).

***Harutaeographa stenoptera* (Staudinger, 1892)**

Holotype: MNHU. Type locality: Ussuri, Amur. Distribution: Russia: (SE Siberia, Amur, Ussuri, Primorje); Korea; China (Shaanxi).

***Harutaeographa yangzisherpani yangzisherpani* Hreblay & Ronkay, 1999**

= *Harutaeographa yangzisherpani transformis* Hreblay & Ronkay, 1999

Holotype: coll. Hreblay, HNHM. Type locality: Thailand, Changwat Chiang Mai, Mts. Doi Inthanon. Distribution: Thailand, Vietnam; Nepal.

## Supplementary Material

XML Treatment for Harutaeographa shui

XML Treatment for Harutaeographa monimalis

XML Treatment for Harutaeographa yangzisherpani transformis

XML Treatment for Harutaeographa pallida

XML Treatment for Harutaeographa cinerea
